# Assessing metabolic properties of dairy cows fed low quality straws by integrative arterial and venous metabolomics

**DOI:** 10.5713/ajas.19.0527

**Published:** 2020-01-13

**Authors:** Bing Wang, Zhu Yu, Jianxin Liu

**Affiliations:** 1 State Key Laboratory of Animal Nutrition, College of Animal Science and Technology, China Agricultural University, Beijing 100193, China; 2 College of Grassland Science and Technology, China Agricultural University, Beijing 100193, China; 3 Institute of Dairy Science, College of Animal Sciences, MoE Key Laboratory of Molecular Animal Nutrition, Zhejiang University, Hangzhou 310058, China

**Keywords:** Corn Stover, Phenylalanine Metabolism, Phenylpropanoate, Metabolomics, Rice Straw

## Abstract

**Objective:**

This study was conducted to reveal potential metabolic differences of dairy cows fed corn stover (CS) and rice straw (RS) instead of alfalfa hay (AH) as main forage source.

**Methods:**

Thirty multiparous mid-late lactation Holstein dairy cows were selected and randomly assigned to three diets, AH, CS, or RS (n = 10). After 13 weeks of the feeding trial, coccygeal arterial and superficial epigastric venous plasma samples were collected before morning feeding for gas chromatography time-of-flight/mass spectrometry analyses.

**Results:**

In the artery, 8 and 13 metabolites were detected as differential metabolites between AH and CS, and between AH and RS, respectively. The relative abundance of phenylpropanoate (log_2_fold change [FC]) = 1.30, 1.09), panthenol (log_2_FC = 2.36, 2.20), threitol (log_2_FC = 1.00, 1.07), and 3,7,12-trihydroxycoprostane (log_2_FC = 0.79, 0.78) were greater in both CS and RS than in AH, and tyrosine (log_2_FC = −0.32), phenylalanine (log_2_FC = −0.30), and pyruvic acid (log_2_FC = −0.30) were lower in RS than in AH. In the vein, 1 and 7 metabolites were detected as differential metabolites between AH and CS, and between AH and RS, respectively. By comparing AH and RS, we found that metabolic pathways of phenylalanine, tyrosine, and tryptophan biosynthesis and phenylalanine metabolism were enriched by integrative artery and vein analysis. Furthermore, AH and RS, arterial phenylpropanoate and 4-hydroxyproline were positively, and phenylalanine was negatively correlated with milk urea nitrogen. Finally, in AH and CS, arterial panthenol was negatively correlated with feed efficiency.

**Conclusion:**

Arterial metabolic profiles changed more than those in the veins from animals on three forage diets, differing in amino acids. We found that phenylalanine, tyrosine, and tryptophan biosynthesis and phenylalanine metabolism were restricted when cows were fed low-quality cereal straw diets.

## INTRODUCTION

Corn stover (CS) and rice straw (RS) are abundant in China but are of low quality [1], both of them are rich in fiber and can be used by ruminants as forage sources [2]. However, reduced milk production and inhibited milk components synthesis indicate that these cereal straws restrict dairy cow production, as shown in our previous studies [3]. We undertook serial studies to identify the underlying mechanism in the mammary gland [4,5]. We found that from the view of lactation physiology [4], phenylalanine metabolism is important for milk synthesis across the mammary gland, and the shortage of leucine and methionine may be a limiting factor for milk protein synthesis when CS or RS is fed to dairy cows [5]. However, the other blood metabolites and metabolic profiles across the mammary had not been clarified for the utilization of cereal straws in dairy cows.

Metabolomics has become an increasingly powerful research field using high-throughput approaches to analyze metabolites of samples with significant molecular complexity, which can be used to identify biological markers and metabolic pathways, offering a better understanding of internal metabolic physiology [6]. Critical metabolic status in dairy cows have been revealed by venous serum metabolomics in determining differences between different quality diets [7,8]. In contrast, Nielsen et al [9] performed arterial plasma metabolomics studies to reveal the differences between different dietary fiber and protein contents in pigs. There is growing evidence that multicomponent analyses of metabolites in arterial plasma can better reflect nutritional metabolic profiles across the mammary glands, such as the use of arterial amino acid (AA) profiles in determining milk protein synthesis [10].

We hypothesized that blood metabolic profiles would be changed after feeding cows cereal straws, which may then result in reduced milk performance. Therefore, the objective of this study was to investigate the differences of arterial and venous metabolome and related metabolic pathways across the mammary gland in cows fed low quality cereal straws instead of alfalfa hay (AH).

## MATERIALS AND METHODS

### Animal ethics statement

The experimental protocols were in accordance with the guidelines and regulations approved by the Animal Care Committee (Approval No. ZJU20130217), Zhejiang University (Hangzhou, China).

### Experimental design and blood sample collection

Thirty multiparous mid-lactation Holstein dairy cows (10 cows per group; body weight = 600±52.0 kg, milk yield = 30.0±3.53 kg, days in milk = 160±27.8 d; and parity = 3.4±1.57; means±standard deviation were assigned to 10 groups based on days in milk and milk production and were randomly allocated to 1 of 3 dietary treatments for this study. The three dietary treatments mainly differed in the forage source. The AH was selected as the high-quality control diet. Two cereal straws (CS and RS) were selected as low-quality forages used to replace AH in the cow feed. Detailed information of the ingredients and nutrient composition of the three diets were shown in our previous study [3]. In brief, the three diets had identical concentrate and corn silage but differed in AH (23%) and Chinese wild ryegrass hay (7%), CS (30%), or RS (30%). The proportions of crude protein in the AH, CS, and RS diets were 16.8%, 16.3%, and 16.2%, respectively. The energy contents in the AH, CS, and RS diets were 6.57, 6.07, and 5.98 MJ/kg respectively. Animals were fed and milked 3 times daily at 06:30, 14:00, and 20:00. Diets were fed as total mixed rations, and orts were collected every day.

After 13 weeks of feeding, two blood samples were collected from the coccygeal artery (arterial sample) and superficial epigastric vein (venous sample) of the 30 cows at 06:00 before the morning feeding and milking by using vacuum tubes (Becton Dickinson, Franklin Lakes, NJ, USA) with lyophilized lithium heparin as an anticoagulant. Blood samples were immediately centrifuged at 3,000×*g* for 15 min at 4°C. Then, the supernatant (plasma) was pipetted and aliquoted (0.5 mL) into appropriately labeled cryovials and frozen at −80°C until the metabolomics analyses were performed.

### Sample preparation and gas chromatography time-of-flight/mass spectrometry analysis

The detailed procedure of sample pre-processing and detection using gas chromatography time-of-flight/mass spectrometry (GC-TOF/MS) is given in our previous study [4].

The GC-TOF/MS analysis was performed using an Agilent 7890 gas chromatograph system coupled with a Pegasus HT TOFMS (LECO, St. Joseph, MI, USA). The system was installed with a DB-5MS capillary column coated with 5% diphenyl cross-linked with 95% dimethyl polysiloxane (30 m×250 μm inner diameter, 0.25 μm film thickness; J & W Scientific, Folsom, CA, USA). A 1-μL aliquot of the analyte was injected in splitless mode. The energy in the electron impact mode was −70 eV. The MS data were acquired at a rate of 20 spectra/s after a solvent delay of 366 s in full-scan mode with a mass-to-charge ratio (m/z) range of 85 to 600. An internal standard (L-2-chlorobenzene alanine) was used to ensure consistency among different GC-TOF/MS runs, which was set as quality control tests. Chroma TOF 4.3X software from the LECO Corporation and the LECO-Fiehn Rtx5 database were used for raw peak extraction, data baseline filtering and calibration of the baseline, peak alignment, deconvolution analysis, peak identification and integration of the peak area following the methods reported by Kind et al [11]. All samples were run in duplicate.

### Identification of different metabolites

The differential metabolites (DMEs) were identified through pairwise comparisons between AH and CS and between AH and RS in arterial and venous plasma. Metabolomics characteristics were analyzed using multivariate statistics by the SIMCA-P^+^13.0 software package (Umetrics, Umea, Sweden). Orthogonal partial least squares discriminant analysis (OPLS-DA) was used to identify the dietary effects on the plasma metabolome. The OPLS-DA model was used to obtain maximal covariance between the measured data and the response variables and to identify the significant DMEs between AH and two cereal straw diets.

To refine the OPLS-DA analysis used to identify the significant DMEs, the first principal component of variable importance projection (VIP) was obtained. Those VIP values exceeding 1.0 were selected first and further considered as changed metabolites. Then, the remaining variables were assessed by Student's T-test. The fold change (FC) value of each metabolite was calculated by comparing the mean value of the peak area obtained from the two groups. The q-value was used to adjust the false discovery rate in the comparison between two diets. If VIP>1, p value<0.05 and |log_2_FC|>0.2, the metabolites were considered to be different between the two groups under comparison. Then, the DMEs were subject to further identification and validation by online database searches, including the Kyoto encyclopedia of genes and genomes and Bovine Metabolome Database.

### Identification of metabolic pathways

To explore potential changes in metabolic pathways under different quality forage diet regulations, identified DMEs were imported into the online analysis platform Metaboanalyst (http://www.metaboanalyst.ca/) following the procedures of Chong et al [12]. The *Bos taurus* (cow) pathway library was used. All matched pathways according to p values from pathway enrichment analysis and pathway impact values from pathway topology analysis were then observed in the metabolome view.

### Correlation analysis

A correlation coefficient (r) and the significance threshold of |r|≥0.53 and p-value<0.01 were applied in the correlation between DMEs and milk performance. The milk performance data were published in our previous study [5]. The correlations were confirmed by scatter plotting of each pair of correlated integrals by using R-3.2.2 statistical software [13].

## RESULTS

### Identification of metabolites and hierarchical cluster analysis

The total number of valid peaks detected in this study was 444. The OPLS-DA plots of the GC-TOF/MS metabolic profiles clearly showed separated clusters in the score plot between AH and CS and between AH and RS in arterial (Supplementary Figure S1) and venous (Supplementary Figure S2) plasma. This suggested that the GC-TOF/MS-based artery and vein metabolomics model can be used to differentiate the two feeding groups.

### Differential metabolites

**Figure 1:**
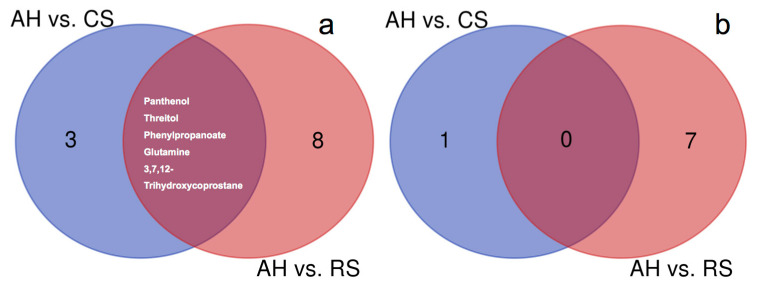
The significantly different metabolites (DME) between AH vs CS and AH vs RS in the artery (a) and vein (b). AH, diet containing alfalfa and Chinese wild rye hay as main forage; CS, diet containing corn stover as main forage; RS, diet containing rice straw as the main forage.

The DMEs between different diets were identified in both arteries (Table 1) and veins (Table 2). For arteries, 8 and 13 DMEs were identified when comparing AH to CS and RS, respectively. Among them, 2-hydroxybutanoic acid (log_2_FC = −0.52) and 2-hydroxyvaleric acid (log_2_FC = −1.01) had lower abundances in CS than in AH, and phenylpropanoate (log_2_FC = 1.30), glutamate (log_2_FC = 0.47), panthenol (log_2_FC = 2.36), allantoic acid (log_2_FC = 0.73), threitol (log_2_FC = 1.00), and 3,7,12-trihydroxycoprostane (log_2_FC = 0.79) had greater abundances in the CS than in AH. The abundances of tyrosine (log_2_FC = −0.32), phenylalanine (log_2_FC = −0.30), pyruvic acid (log_2_FC = −0.30), and 5-aminovaleric acid (log_2_FC = −0.67) were lower in RS than in AH. The abundances of 4-hydroxyproline (log_2_FC = 0.63), galacturonic acid (log_2_FC = 0.68), phenylpropanoate (log_2_FC = 1.09), p-cresol (log_2_FC = 0.61), glutamate (log_2_FC = 0.40), panthenol (log_2_FC = 2.20), prostaglandin A2 (log_2_FC = 18.39), threitol (log_2_FC = 1.07), 3,7,12-trihydroxycoprostane (log_2_FC = 1.36) and ornithine (log_2_FC = 1.36) were greater in RS than in AH. The mutual DMEs in the two dietary comparisons in the arterial plasma were panthenol, threitol, phenylpropanoate, glutamate, and 3,7,12-trihydroxycoprostane (Figure 1a).

In venous plasma, 1 and 7 DMEs were identified in the comparisons between AH and CS and AH and RS, respectively. The abundance of phenylpropanoate (log_2_FC = 1.17) was greater in CS than in AH. The abundances of valine (log_2_FC = −0.52), phenylalanine (log_2_FC = −0.46), and pyruvic acid (log_2_FC = −0.41) were lower in RS than in AH. The abundances of creatine (log_2_FC = 0.46), 4-hydroxyproline (log_2_FC = 0.77), benzoic acid (log_2_FC = 1.15), and 3,7,12-trihydroxycoprostane (log_2_FC = 0.89) were greater in RS than in AH. No mutual DMEs were found by comparing AH and CS and AH and RS (Figure 1b).

### Metabolic pathway

**Figure 2:**
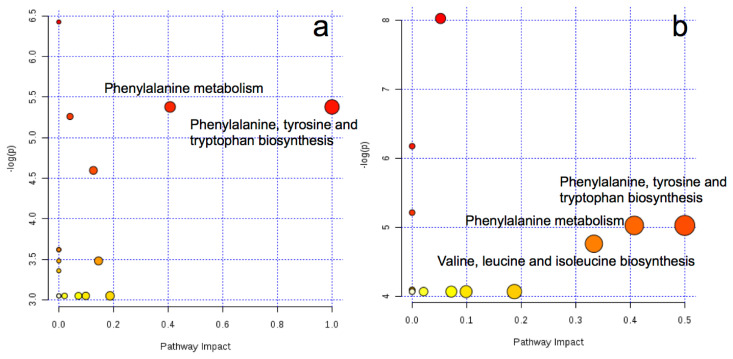
Metabolome view map of the differential metabolites identified from the comparison between cows fed AH and RS based diets in the artery (a) and vein (b). Larger sizes and darker colors represent higher pathway enrichment and higher pathway impact values, respectively. The x-axis represents the pathway impact and y-axis [−log(p-value)] represents pathway enrichment. AH, diet containing alfalfa and Chinese wild rye hay as main forage; RS, diet containing rice straw as the main forage. Metaboanalyst (http://www.metaboanalyst.ca) generated topology map described the impact of baseline metabolites identified between high quality forage diet (AH) vs low quality forage diet (RS) groups with high VIP values (VIP>1) on metabolic pathways. The *Bos taurus* (cow) pathway library was used.

The key metabolic pathways are shown in Figure 2. By comparing between AH and RS, we found the two key different metabolic pathways in arterial plasma, i.e., phenylalanine, tyrosine, and tryptophan biosynthesis and phenylalanine metabolism. Similarly, the three key different metabolic pathways in venous plasma were identified as phenylalanine, tyrosine, and tryptophan biosynthesis; phenylalanine metabolism; and valine, leucine and isoleucine biosynthesis. No significantly enriched metabolic pathways were found in the comparison between AH and CS in either arterial or venous plasma.

### Relationship between milk performance and arterial differential metabolites between diets

**Figure 3:**
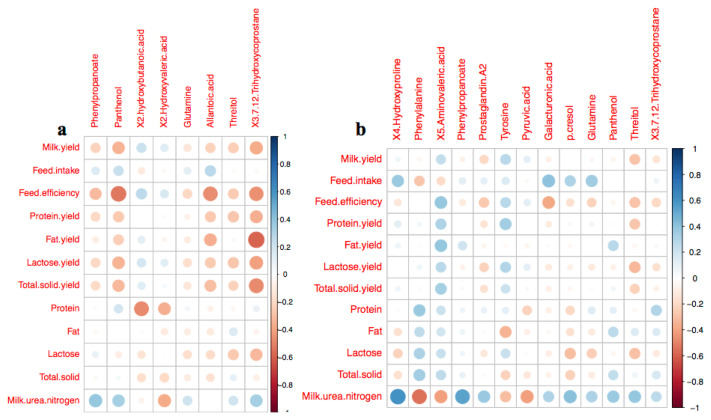
Heatmap of the linear correlation between the characteristics of milk performance and the abundance of arterial differential metabolites based on the comparison between AH and CS (a) and the comparison between AH and RS (b). The circle size was correlated with the color. The deeper red color, the higher negative correlation; the deeper blue color, the higher positive correlation. The bigger circle size means the deeper color. The circle size relates with the absolute value of correlation (|R|). The bigger circle size, the higher correlation. AH, diet containing alfalfa and Chinese wild rye hay as main forage; CS, diet containing corn stover as main forage; RS, diet containing rice straw as the main forage.

The relationship between the arterial DMEs and selected milk performance characteristics on the three different diets was further subjected to a correlation analysis by using linear regression models (Figure 3). Based on the DMEs from AH and CS, the abundance of arterial panthenol was negatively correlated with feed efficiency (r = −0.53, p<0.05), and arterial 3,7,12-trihydroxycoprostane was negatively correlated with milk fat yield (r = −0.58, p<0.05). In DMEs from AH and RS, the abundances of arterial 4-hydroxyproline (r = 0.59, p<0.05) and phenylpropanoate (r = 0.53, p<0.05) were positively correlated with milk urea nitrogen (MUN) content, but phenylalanine was negatively correlated with MUN content (r = −0.54, p<0.05).

## DISCUSSION

**Figure 4:**
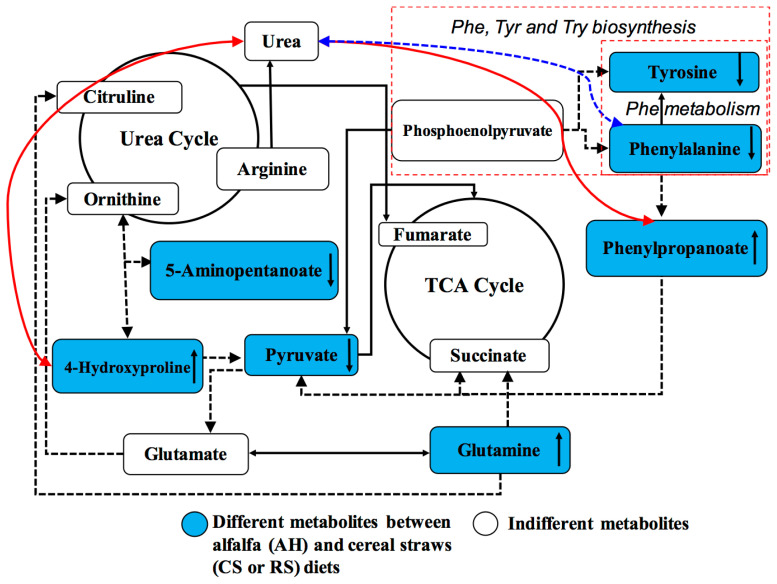
A proposed working hypothesis of the nutritional disadvantage of cereal straws (CS and RS) fed to dairy cows based on the key differential metabolites between the two cereal straws and AH based diets. The metabolites under the blue color box denote the significantly different between AH diet and cereal straws diets (CS or RS). Arrows upward indicate greater abundance in CS or RS diet than in AH diet. Arrows in downward direction indicate the lower abundance in CS or RS diet than in AH diet. Arrows with a straight solid line indicate a direct process between the two metabolites. Arrows with straight dotted lines indicate an indirect process (more than one step) between the two metabolites. Arrows with red curved solid lines indicate a significantly positive correlation between the two items whereas arrows with blue curving dotted line indicate significantly negative correlations between the two items. Phe, phenylalanine; Tyr, tyrosine; Trp, tryptophan; AH, diet containing alfalfa and Chinese wild rye hay as main forage; CS, diet containing corn stover as main forage; RS, diet containing rice straw as the main forage.

The identification of blood DMEs from cows on different diets provides a useful approach to explore the key metabolic processes associated with dietary effects [7,9]. The current study aimed at profiling all small metabolic molecules based on integrative arterial and venous plasma metabolomics in dairy cows, which could help us to identify new, key metabolites in dietary nutrient metabolism. By linking dietary composition with arterial and venous metabolomes together, the effects of low-quality cereal straw feeding on blood metabolism, key metabolites, and the underlying metabolic networks were observed (Figure 4).

The low-quality forage diets (CS and RS) largely inhibited the activity of AA metabolism but improved the urea cycle in dairy cows, based on DMEs (Figure 4). The current findings of decreased tyrosine, valine, phenylalanine and pyruvic acid were consistent with the dietary differences and the improved MUN concentration in cows fed RS compared to AH [3]. For MUN content, arterial phenylalanine was shown to be highly positively correlated, but arterial 4-hydroxyproline and phenylpropanoate were negatively correlated (Figure 4). Free blood-derived 4-hydroxyproline is generated from the degradation of collagens or other proteins containing 4-hydroxylprolyl residues [14]. Phenylpropanoate is the indirect catabolite of phenylalanine [15]. Thus, the positive correlation between these two metabolites and MUN might confirm that protein or AA degradation in cows would result in increased MUN in milk. In addition, these findings confirmed restricted AA and glucose metabolism after cows were fed low-quality forage diets (CS and RS), as shown in our previous studies [5,16]. Sun et al [8] found that cows fed a low-quality CS diet showed changes in the glycine, serine, and threonine metabolism, tyrosine metabolism, and phenylalanine metabolism pathways, which are similar to the results of current study. The important pathways based on identified DMEs between AH and cereal straw diets were mainly related to phenylalanine, tyrosine, and tryptophan biosynthesis; phenylalanine metabolism; and valine, leucine and isoleucine biosynthesis. Phenylalanine, tyrosine, and tryptophan biosynthesis and valine, leucine and isoleucine biosynthesis do not exist in mammary glands [17], but this process may indicate the limited AA anabolism and AA redistribution in the liver of cows fed cereal straws. The DMEs of phenylalanine, tyrosine, and valine enriched in these metabolic pathways decreased after cows were fed cereal straw diets, indicating that a shortage of these AAs was the limiting factor in the lactating cows fed cereal straws. These AAs are the key milk protein precursors in dairy cows [18], and their shortage in arterial supply would then result in decreased mammary uptake [10]. Thus, the decreased levels of these AAs in arterial plasma might result in restricted milk protein synthesis. In addition, the shortage of valine in venous plasma was consistent with our previous study using quantitative analysis of AA [5]. In the current study, the arterial glutamate concentration was decreased after cows were fed CS and RS compared to AH, but it did not decrease in the vein. This finding is different from the results of our previous study in which we found the similar arterial glutamate concentration between the three diets [5]. Glutamate is the main AA precursor and primarily acts as a nitrogen donor for other AAs [19]. A high demand for glutamate for protein synthesis has been reported in the mammary gland [20,21], which is able to generate a large arteriovenous difference in glutamate [21]. The increased arterial glutamate along with decreased essential AAs concentrations in CS-fed cows confirmed the shortage of essential AAs, which then required more glutamate uptake by the mammary gland for the transaminase process [10]. Thus, our results confirmed the function of glutamate as an AA precursor and a nitrogen donor.

The lower abundance of arterial pyruvic acid in RS group might reflect the lack of energy supply of the RS diet compared to the AH diet, which is consistent with restricted gluconeogenesis in the liver and glucose metabolism in the mammary glands [16]. In addition, arterial phenylpropanoate in cows fed CS or RS was much greater than that in cows fed AH, and prostaglandin A2 was only found in the RS diet. Phenylpropanoate is a decomposed metabolite of phenylalanine; thus, the greater arterial phenylpropanoate in cows fed CS and RS may indicate the shortage of energy or other nonessential AAs from CS and RS diets that require the decomposition of phenylalanine [22]. Prostaglandin A is derived from arachidonic acid and has an anti-proliferative function by regulating the expression of apoptosis genes [23]. Thus, the generation of prostaglandin A in cows fed RS may restrict mammary cell proliferation, which would decrease the number of mammary cells [24]. In addition, in mammals, prostaglandins play important roles in several physiological and pathological processes. For example, they have been shown to induce chemokines resulting in infiltration of inflammatory cells, such as eosinophils, neutrophils, and macrophages [25], which are important for consequent adaptive immunity [26]. Therefore, the greater prostaglandin A secretion in cows fed RS might indicate that the mammary glands of RS group cows might have had a certain inflammatory response. A previous study also reported that urea at a high concentration could stimulate the over-secretion of prostaglandin E2 in dairy cows [27]. Thus, the greater abundance of prostaglandin A in the arteries of cows fed RS might be attributed to the lower nitrogen utilization efficiency, which was revealed in our previous study [3]. However, we could not explain why cows fed with CS diet did not have prostaglandin A2. More studies will be conducted in the future to determine the underlying mechanism.

A much greater number of DMEs were found in the artery than the vein in the comparisons between AH and cereal straw diets, indicating the important role of arterial blood in the study of nutrient metabolism in the mammary glands. Previous studies have reported that there are certain differences between arteries and veins with regard to metabolic activity [28]. The vein is always selected as sampling item in dairy cows’ studies [29]. However, the artery is more sensitive to reflect the AA metabolism in dairy cows than vein [10]. Thus, the results based on arterial and venous metabolomics from this study confirmed that the artery is very effective in identifying dietary differences. On the other hand, most of the DMEs identified in our study showed a higher q-value above 0.05. This might be mainly due to the insufficient number of N = X cows used in each group. Thus, more replicates should be included in future studies in analyzing plasma metabolome. In addition, another limitation of our study is that the blood was sampled in the morning before feeding, which may have affected the metabolites obtained in our study. Therefore, further study should address the effects of sampling time on the changes of plasma metabolome in dairy cows.

## CONCLUSION

Arterial plasma was shown to have a pronounced effect reflecting dietary differences and is therefore a good candidate to study diet difference. Phenylpropanoate and 4-hydroxyproline were positively related to MUN, but phenylalanine was negatively related to MUN, indicating their roles in affecting N metabolism in cows. Arterial panthenol was negatively related to feed efficiency. Our results revealed that the increased level of arterial phenylpropanoate and 4-hydroxyproline and the decreased level of phenylalanine may be the crucial factors when cows were fed low-quality cereal straw diets. The AA metabolism (phenylalanine, tyrosine and tryptophan biosynthesis, phenylalanine metabolism) pathways were largely impaired when cows were fed cereal straw diets, accounting for the different dietary nutritional effects.

## Figures and Tables

**Table 1:** Identification of significantly different arterial metabolites between the cows fed three different diets

Metabolites name1)	Similarity	RT	Mass	VIP	p-value	q-value	log_2_FC
AH vs CS							
2-Hydroxybutanoic acid	816	8.25	131	2.27	0.046	0.57	−0.52
2-Hydroxyvaleric acid	694	9.45	131	2.50	0.04	0.56	−1.01
Phenylpropanoate	626	12.34	104	2.00	<0.01	<0.01	1.30
Glutamate	590	16.46	156	1.90	0.03	0.51	0.47
Panthenol	585	17.35	105	1.40	<0.01	<0.01	2.36
Allantoic acid	575	19.52	259	1.00	0.04	0.54	0.73
Threitol	505	13.30	103	1.80	0.03	0.50	1.00
3,7,12-Trihydroxycoprostane	501	27.78	129	1.94	0.03	0.52	0.79
AH vs RS							
Tyrosine	914	18.19	218	1.83	0.03	0.45	−0.32
4-Hydroxyproline	858	13.64	140	2.43	<0.01	0.13	0.63
Phenylalanine	828	14.86	218	2.42	<0.01	0.13	−0.30
Pyruvic acid	798	7.14	174	1.67	0.04	0.49	−0.30
Galacturonic acid	743	18.12	333	1.12	<0.01	0.22	0.68
5-Aminovaleric acid	743	14.87	174	1.09	<0.01	0.13	−0.67
Phenylpropanoate	626	12.34	104	1.25	<0.01	0.07	1.09
p-cresol	601	8.69	165	1.08	0.03	0.46	0.61
Glutamate	590	16.46	156	1.89	0.03	0.46	0.40
Panthenol	585	17.35	105	2.07	0.01	0.29	2.20
Prostaglandin A2	564	24.62	105	2.78	<0.01	0.14	18.39
Threitol	505	13.30	103	1.70	0.03	0.47	1.07
3,7,12-Trihydroxycoprostane	501	27.78	129	1.85	0.047	0.51	0.78

RT, retention time; VIP, variable importance projection; q-value, the false discovery rate; FC, fold change (the later/the former, etc. AH vs. CS, FC = CS/AH).

1) AH, diet containing alfalfa and Chinese wild rye hay as main forage; CS, diet containing corn stover as main forage; RS, diet containing rice straw as the main forage.

**Table 2:** Identification of significantly different venous metabolites between the cows fed three different diets

Metabolites name	Similarity	RT	Mass	VIP	p-value	q-value	log_2_FC
AH vs CS							
Phenylpropanoate	626	12.34	104	1.54	<0.01	<0.01	1.17
AH vs RS							
Creatine	890	13.99	115	5.25	0.02	0.35	0.46
4-Hydroxyproline	858	13.64	140	1.00	<0.01	0.03	0.77
Valine	846	9.54	144	7.35	0.01	0.28	−0.52
Phenylalanine	828	14.86	218	1.20	<0.01	0.25	−0.46
Pyruvic acid	798	7.14	174	1.27	0.02	0.28	−0.41
Benzoic acid	698	10.04	179	1.59	<0.01	0.17	1.15
3,7,12-Trihydroxycoprostane	501	27.78	129	4.67	0.03	0.44	0.89

RT, retention time; VIP, variable importance projection; q-value, the false discovery rate; FC, fold change (the later/the former, etc. AH vs CS, FC = CS/AH).

1) AH, diet containing alfalfa and Chinese wild rye hay as main forage; CS, diet containing corn stover as main forage; RS, diet containing rice straw as the main forage.
