# Supplementary material for: Assessing metabolic properties of dairy cows fed low quality straws by integrative arterial and venous metabolomics
Source: Asian-Australas J Anim Sci. 2020 Jan 13;33(11):1770–8. doi: 10.5713/ajas.19.0527 (PMC7649076; doi:10.5713/ajas.19.0527)
Supplement: Supplementary file 1 [file ajas-19-0527-suppl.pdf]

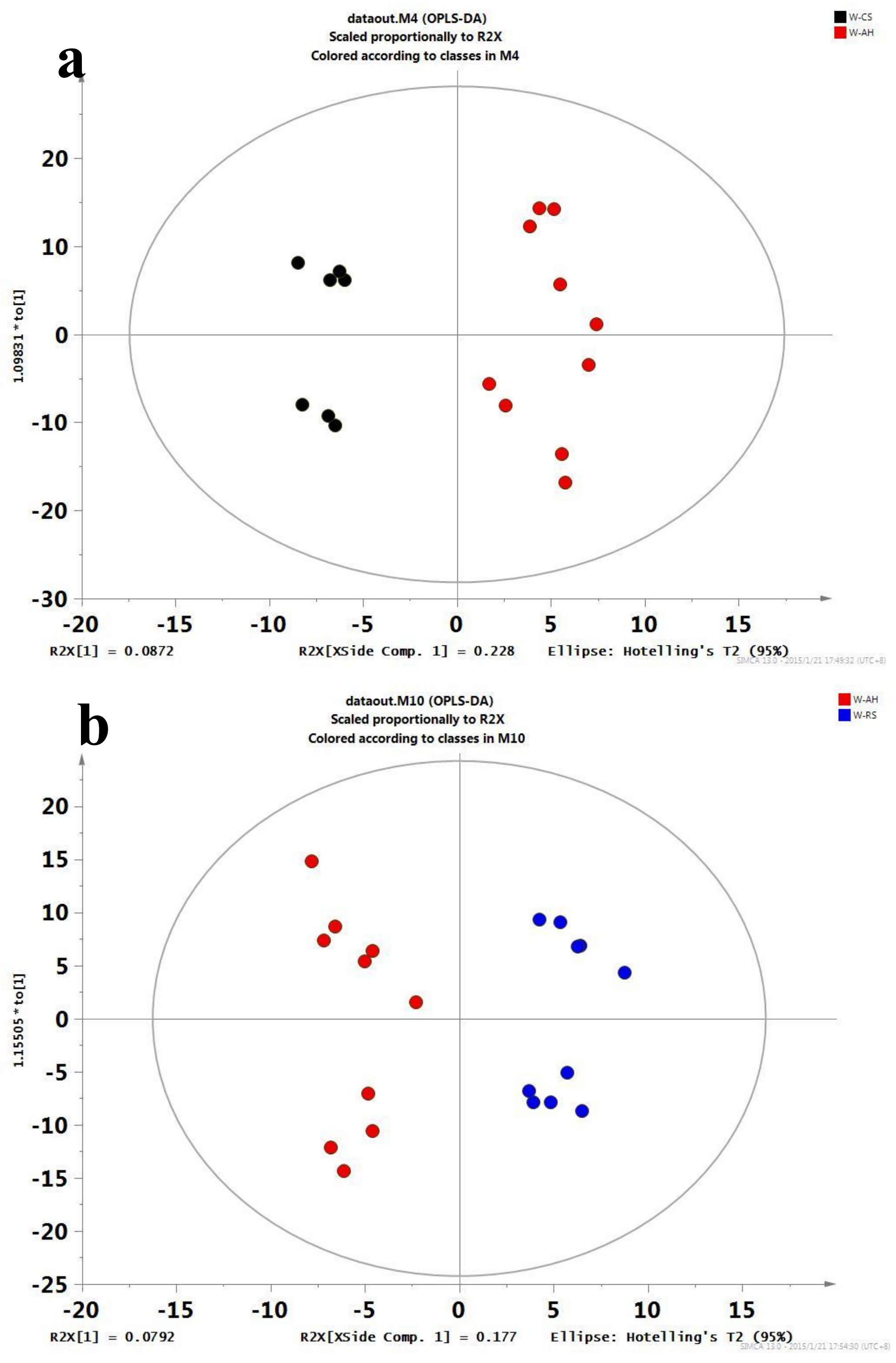

Figure S1. Score plot of orthogonal partial least squares discriminant analysis model obtained of CS and AH (a), RS and AH (b) in artery. AH = diet containing alfalfa and Chinese wild rye hay as main forage; CS = diet containing corn stover as main forage; RS = diet containing rice straw as the main forage.

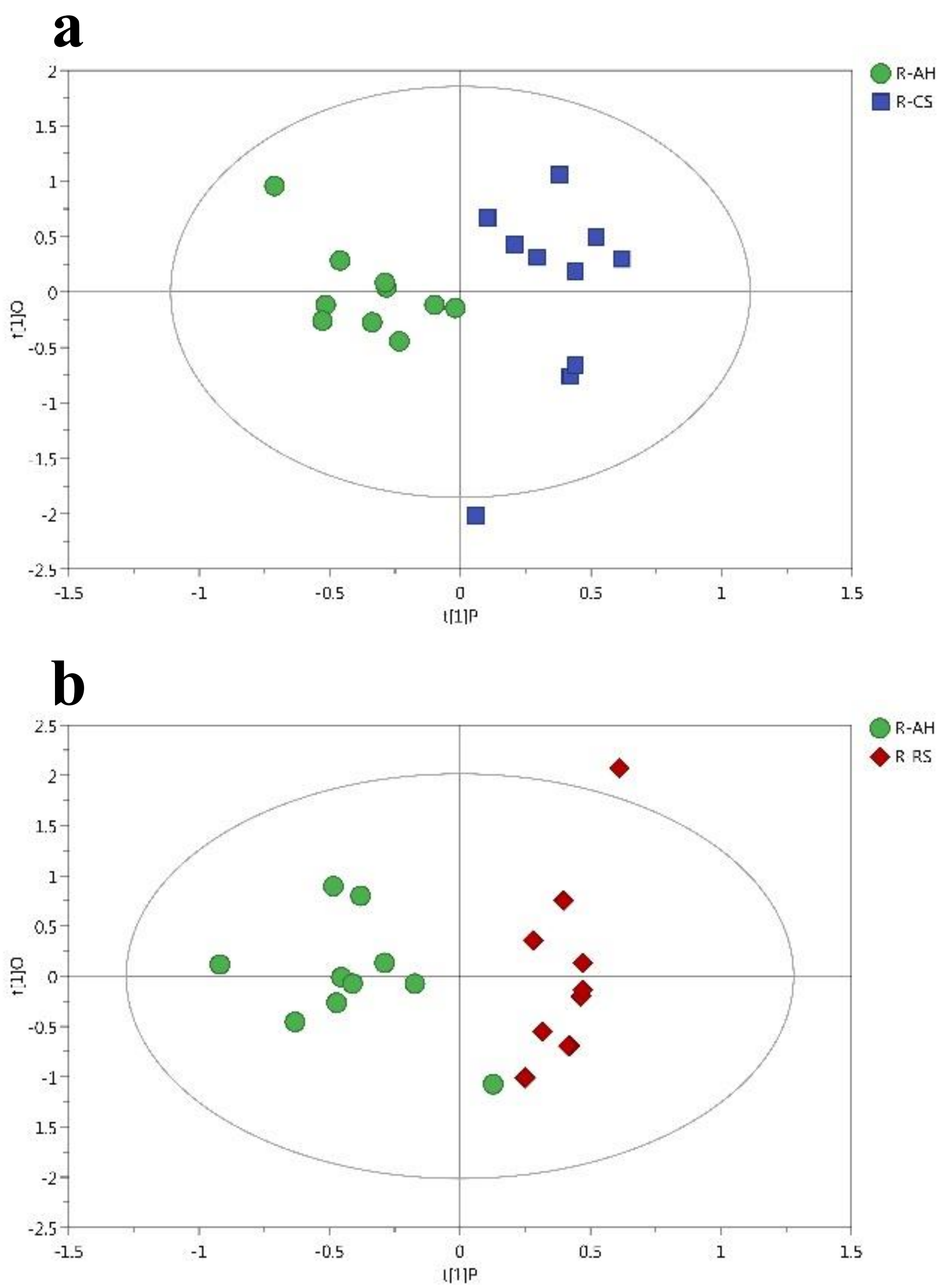

Figure S2. Score plot of orthogonal partial least squares discriminant analysis model obtained of CS and AH (a), RS and AH (b) in vein. AH = diet containing alfalfa and Chinese wild rye hay as main forage; CS = diet containing corn stover as main forage; RS = diet containing rice straw as the main forage.
